# How do tissue infiltrating B cells and plasma cells correlate with other inflammatory features in muscle tissue from patients with JDM?

**DOI:** 10.1186/1546-0096-12-S1-P87

**Published:** 2014-09-17

**Authors:** Erdal Sag, Shireena A Yasin, Katie Arnold, Janice L Holton, Tom S Jacques, Lucy R Wedderburn

**Affiliations:** 1 Infection, Inflammation and Rheumatology Unit, Institute of Child Health, University College London, UK; 2 Department of Molecular Neuroscience, MRC Centre for Neuromuscular Diseases, Institute of Neurology, University College London, UK; 3 Birth Defects Research Centre, Institute of Child Health, University College London, London, UK

## Introduction

The International Juvenile Dermatomyositis (JDM) Biopsy Consensus Group has previously published and validated a scoring tool for assessment of the severity of pathological change in biopsy specimens from patients with suspected or proven JDM. This tool assesses histological features characteristic of JDM, organised into four domains (inflammatory, vascular, muscle fibre and connective tissue). The score tool includes the assessment of CD3+ and CD68+ inflammatory cells. Given that JDM is a disease characterised by production of autoantibodies and some patients respond to anti-B cell therapy we hypothesise that B cells play an important role in the pathogenesis of the disease.

## Objectives

We aimed to define the extent and patterns of B cell and plasma cell infiltration in JDM muscle biopsy tissue and compare these features with inflammatory domain scores of the validated score tool.

## Methods

Muscle biopsies from the UK Juvenile Dermatomyositis Cohort and Biomarker Study taken at the time of disease presentation were analysed. All children had definite or probable JDM according to the Bohan and Peter criteria, disease duration of <12 months before biopsy and had their biopsy sample taken before use of steroids or disease-modifying agents such as methotrexate or other immunosuppressive agents. Each biopsy was stained for cells expressing CD20 (B cells), CD138 (plasma cells), CD3 (T cells), and CD68 (monocytes/macrophages). For each of endomysial, perimysial and perivascular distributions, scoring was performed for CD3+, CD68+, CD20+ and CD138+ infiltrating cells using the criteria of the score tool. Spearman’s rank correlation coefficient was used to assess correlation between score data elements. SPSS 21.0 was used for statistical analysis.

## Results

Twenty-six patients with JDM (14 male, 12 female) were included in this study. 73% of biopsies (n=19) contained CD20+ B cells while only 26% of biopsies (n=7) contained CD138+ plasma cells. The score for CD20+ cells was strongly correlated with the score for CD3+ cells (r=0.81; p<0.0001) and the inflammatory domain score (r=0.87; p<0.0001). Among those biopsies that contained CD138+ plasma cells, the CD138+ score was correlated with the score for CD20+ cells (r=0.89; p=0.026), the score for CD3+ infiltrating cells (r=1.0; p<0.0001) and the inflammatory domain score (r=0.84; p=0.015). In most cases, B cells were co-localised with T cells especially at perivascular and endomysial regions but in some cases they were diffusely scattered. No specific patterns were observed for plasma cells which were found as individual scattered cells mainly in the perimysium.

## Conclusion

Both B cells and plasma cells are present in the inflamed tissue of some muscle biopsies of JDM patients. In this cohort of JDM patients, B cell and plasma cell infiltration was correlated with CD3+ cells and the inflammatory domain scores of the published score tool. The distinct patterns of B and plasma cell infiltration and how these correlate with autoantibody production and clinical features, warrant further investigation.

## Disclosure of interest

None declared.

